# Performance Evolution and Damage Evaluation of CRTS I Track Slab in Service Status

**DOI:** 10.3390/ma18092041

**Published:** 2025-04-29

**Authors:** Hongyao Lu, Wentao Wu, Yuelei He

**Affiliations:** 1 College of Urban Rail Transportation, Shanghai University of Engineering Science, Shanghai 201600, China; m405123413@sues.edu.cn (W.W.); hyldoc@163.com (Y.H.); 2 Shanghai Key Laboratory of Structural Durability and System Safety of Rail Transit, Tongji University, Shanghai 201800, China

**Keywords:** ballastless track slab, void coefficient, performance evolution, load combination, damage assessment

## Abstract

This study develops a quantitative framework to assess performance degradation and damage evolution in CRTS I ballastless track slabs. Based on the impact-echo method, the internal void distribution characteristics of the new and old track slabs were obtained. The track slabs were sampled separately by drilling cores to verify the distribution of voids, and uniaxial compression tests were conducted simultaneously to quantify the attenuation of bearing capacity. The on-site wheel–rail force and temperature field data were monitored, based on the established three-dimensional finite element model of CRTS I ballastless track, and the damage distribution characteristics of the track slab under different load combinations after performance degradation were studied. The results show the following: (1) As the performance of the track slabs gradually deteriorated, it was reflected in the increasing internal void distribution area from 0.5% to 3.6%, corresponding to a 22.4% decrease in core strength. (2) The on-site monitoring results showed that the average wheel–rail force was 84.5 kN. The temperature gradient range varied from −50.4 °C/m to 100.0 °C/m, exceeding the allowable value of the design specifications. (3) The actual damage distribution of the track slab after performance degradation under different load combinations significantly increased at key stress locations such as near fasteners, convex abutments, and anchor holes of prestressed steel bars, which required special attention in actual maintenance and repair.

## 1. Introduction

The ballastless track system used in high-speed railways replaces the traditional road ballast bed with a monolithic foundation composed of materials such as concrete and asphalt mixtures. This system offers advantages such as high stability, durability, and reduced maintenance requirements, and it has been widely applied in China’s railway construction, particularly for high-speed railways. At present, China’s high-speed railway has transitioned from large-scale construction to long-term safe and stable operation. A critical challenge lies in developing scientific approaches to maintain the operational integrity of high-speed railway lines, ensuring their safety and reliability over the long term. Under the combined effects of dynamic loads from trains and temperature variations, the microstructure of the materials comprising the ballastless track slab undergoes changes during its long service life, which can lead to damage or even failure in critical components or areas. The continued degradation of the ballastless track slab’s performance during service inevitably results in the propagation of damage on the surface and within the track structure, posing a threat to the safety of high-speed trains and significantly reducing the durability of the track. Therefore, conducting theoretical and experimental research on the damage evolution of ballastless track slabs under service conditions is crucial for quantifying the actual service performance degradation and evaluating the damage state of the track slabs.

Regarding the stress forms and damage characteristics of ballastless tracks in service, scholars typically employ testing and simulation analyses. These studies measure the temperature and train loads acting on the track slab structure [1,2,3,4,5], followed by an analysis of the slab stress characteristics and fatigue mechanisms under different load conditions and boundary constraints [6,7,8]. As a typical layered structure system, the track slab is subjected to the combined effects of overall temperature changes and temperature gradients. Simultaneously, it undergoes repeated high-frequency train loads [9], which can easily result in slab deformation [10,11,12,13,14,15]. In the assessment of concrete under actual service conditions, various Structural Health Monitoring (SHM) methods are employed. Surface crack propagation is commonly monitored using image-based techniques, infrared thermography, and acoustic emission methods [16,17,18,19,20]. In addition, internal void detection is typically performed using impact-echo and ground penetrating radar methods [21,22,23], while core strength testing at critical locations provides further insight into material degradation [24,25,26].

The limited research on the residual bearing capacity of ballastless tracks under fatigue loading has primarily focused on the final failure modes [27,28], with little exploration of the calculation and degradation patterns of residual bearing capacity at different stages during service. Most existing studies treat ballastless tracks as homogeneous continuous media, and macroscopic models fail to reveal the relationship between internal damage within the track slab and its macroscopic mechanical properties, making it difficult to reasonably explain the damage propagation patterns and failure mechanisms. Additionally, once damage occurs in the track slab, the load-bearing and transmission characteristics of the structure are inevitably altered [29,30,31,32,33]. However, studies on the location of damage and its propagation patterns under identical loading conditions remain unclear, highlighting the urgent need for further research into the damage evolution of ballastless track slabs.

Based on the aforementioned research background and scientific issues, this paper employed the impact-echo method to statistically analyze internal voids in new, unused track slabs and in damaged ballastless track slabs that have been in service for 10 years. Additionally, core sampling was used to calculate the actual compressive bearing capacity of both new and old track slabs. A correlation between the residual bearing capacity of the track slabs and the void ratio was further established. Using the concrete plastic damage theory and stochastic pore theory, a finite element model (FEM) was developed to analyze the condition of ballastless track slabs under damage scenarios. The monitoring of temperature and wheel–rail forces on actual track slabs was conducted to obtain the load characteristics during service, which were then used as key parameters for the FEM analysis. This study quantified the damage states of track slabs under different working conditions, clarified how damage alters the load transfer paths and the propagation of damage, and provided significant insights into the scientific quantification of the actual service condition of ballastless tracks.

## 2. Track Slab Damage State Quantification

The CRTS I slab track is a precast concrete structure composed of cement, sand, aggregates, and water. However, due to the inherent limitations of the casting and vibrating process, small internal voids and defects are present. Changes in the microstructural parameters directly affect the macroscopic mechanical properties of the concrete, such as its elastic modulus and compressive strength. The issues of cracking and spalling under fatigue loads are the result of accumulated damage in the track slab. Quantifying the distribution and evolution of internal micropores before the track slab experiences cracking and failure, and obtaining the pore parameters, can clarify the impact of these microstructural features on the slab’s macroscopic properties. Furthermore, establishing the relationship between the void ratio and concrete damage will help define the residual load-bearing capacity of the structure.

Compared to new track slabs, the bearing capacity and mechanical properties of those that have been in service for years degrade significantly. In this study, both new and old track slabs (the latter having been in service for 10 years) were examined as research objects. Impact-echo testing and core sampling were performed on both new and old slabs to assess their damage states. The internal structural damage of both slab types was evaluated using the impact-echo and imaging methods, and the internal void coefficients of the new and old slabs were quantified. Additionally, core drilling and uniaxial compression tests were conducted to measure the compressive strength of the components. The attenuation of bearing capacity and the failure characteristics of both new and old slabs were further analyzed.

### 2.1. Method for Determining the Emptying Coefficient of the Track Slab

**Figure 1:**
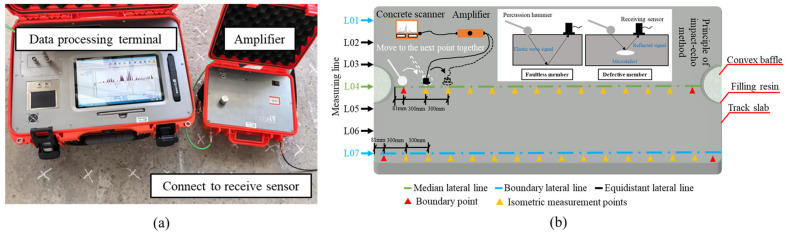
Percussion experiment with impact-echo method. (**a**) Impact-echo method equipment. (**b**) The principle of the impact-echo method and the design of the test scheme of track plate emptying.

Based on the impact-echo method, elastic wave signals are continuously generated along the surface of the track slab. When there are voids within the slab, the significant difference in mechanical impedance between air and concrete causes nearly all of the elastic waves to be reflected back into the concrete. After multiple reflections, the elastic wave signals eventually reach the surface receiver on the track slab. Using Fourier transform, the time-domain signals are converted into frequency-domain signals, from which the thickness of the concrete structure or the depth of the defect can be calculated. Through signal analysis, the relationship between the signals and the defects can be identified, allowing for a clear assessment of the internal voids within the track slab. The impact-echo test setup is illustrated in Figure 1.

As shown in Figure 1a, the impact-echo testing equipment consists of a data processing terminal, an amplifier, receiving sensors, and excitation devices. The experimental scheme for detecting internal voids in the track slab using the impact-echo method is illustrated in Figure 1b. Measurement lines were arranged longitudinally along the track slab, while transverse measuring lines 01–07 were evenly spaced at intervals of 300 mm across the slab. Lines 01 and 07, designated as boundary lines, were positioned 150 mm away from the slab edges, while line 04 was the central measuring line. Measurement points were distributed along the measuring lines at equal intervals of 300 mm. Due to dimensional constraints of the track slab, boundary measurement points were located 81 mm from the slab ends. Additionally, due to the slab design, which included reserved spaces for protruding end stops and filling resin, the central measuring line (line 04) contained two fewer points than the other measuring lines.

During the experiment, the track slab was placed on wooden sleepers to ensure effective elastic wave reflection within the slab. Prior to testing, wave velocity calibration was conducted, and subsequent void ratio measurements of new and old track slabs were performed based on the measurement point arrangement. In the testing process, the excitation hammer and receiving sensor were placed on adjacent points along the same measuring line. The excitation hammer was used to strike the measurement points, while the receiving sensor captured the reflected signals at the adjacent points. After completing the test at one point, both the excitation hammer and receiving sensor were moved simultaneously to the next point, following a “move-and-test” procedure. The impact force was meticulously controlled to ensure sufficient wave reflection while avoiding damage to the concrete surface. Each measurement point was tested three times to ensure data reliability and consistency.

### 2.2. Core Sampling to Measure the Remaining Bearing Capacity of the Track Slab

**Figure 2:**
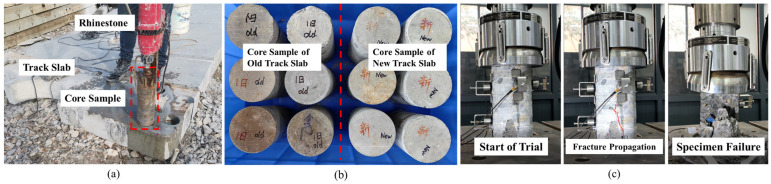
Uniaxial compression test of cylinder specimen. (**a**) Core sampling at key position of track slab. (**b**) Track slab core samples. (**c**) Damage process of core sample.

When using the core drilling machine (Beijing Zhongjiao Jianyi Technology Development Co., Ltd., Beijing, China) to sample the new and old track slabs on site, the machine should be smoothly positioned on the surface of the track slab and securely fixed in place. The cooling water flow rate should be adjusted to 3–5 L/min, which serves to cool the high temperatures generated by friction between the drill bit and the track slab surface during drilling, as well as to wash away concrete debris. The core samples extracted from the track slabs are cylindrical specimens with a diameter of 100 mm and a height of 190 mm. Prior to conducting the uniaxial compression test on the cylindrical specimens, the surfaces of the samples should be thoroughly cleaned, and both the upper and lower surfaces should be polished using a grinding machine (Beijing Laituo High-tech Instruments Co., Ltd., Beijing, China) to ensure that they are flat and smooth. The uniaxial compression test was performed on the cleaned core specimens from the track slabs. Six core samples from both the new and old track slabs were selected and placed sequentially onto the loading platform of a universal tensile and compression testing machine (Jinan Dongce Testing Machine Technology Co., Ltd., Jinan, China). Before starting the test, we ensured that the upper and lower surfaces of each specimen were in close contact with both the loading head of the testing machine and the base platform. The geometric parameters of the specimen and the loading parameters for the test were defined, with the loading mode set to force-controlled and the loading rate specified as 5.0 kN/s. The core testing procedure is illustrated in Figure 2.

### 2.3. The Correspondence Between the Unloading Coefficient of Track Slab and the Remaining Bearing Capacity

In order to evaluate the internal damage state of the track slab that has been in service for many years, the impact-echo method was used to carry out the void coefficient test of the track slab with the new and old track slabs as the research object, and the unloading cloud map of the internal structure of the new and old track slabs was, respectively, detected and obtained. The quantitative results of the internal unloading of the new and old track slabs were verified based on the image method and the concrete interface unloading map of the drill core sample.

**Figure 3:**
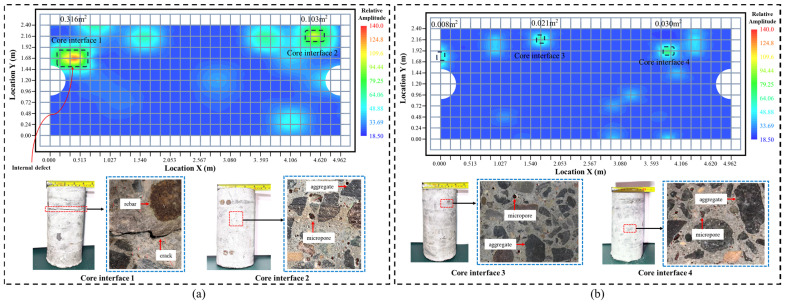
Cloud image of inner cavity of track slab and local cavity image of drill core sample. (**a**) Used slab apparent strength and drill core diagram. (**b**) New slab apparent strength and core drilling diagram.

Based on the internal damage and void contour maps of both new and old track slabs, it can be observed that the void ratio of the unused new track slab is only 0.5%, with an overall “point-like” void distribution and relatively small localized void areas; the void cloud map and drilled core cross-sectional image of the unused new slab are shown in Figure 3b. In contrast, the void ratio of the 10-year-old track slab in service reaches 3.6%, with a more continuous “block-like” void distribution, indicating larger and more connected void areas; the void cloud map and drilled core cross-sectional image of the used old slab are shown in Figure 3a. According to the local void maps from core drilling samples, the new slab contains a few microvoids, predominantly located at the interface between aggregate and cement. In the old slab, the number of microvoids increases significantly, with many microvoids connected by microcracks and, in some cases, even leading to the formation of macroscopic interfacial cracks. Using the impact-echo method, the differences in the internal void ratios between new and aged track slabs were quantitatively characterized. Subsequently, based on the experimental data, 0.5% and 3.6% of the mesh elements were removed from the finite element model to represent the void ratio of the new and aged track slabs, respectively.

The uniaxial compression test on core samples extracted from both new and old track slabs provides the load–displacement curve of the components. Based on the cross-sectional area and peak load of the components, the compressive strength of the core samples can be calculated. In accordance with the Technical Code for Testing Concrete Strength Using Core Drilling Method [34], the compressive strength of the core samples can be converted into the equivalent compressive strength of a cubic specimen with a side length of 150 mm, tested at the same age. This allows for the determination of the compressive strength of the concrete in both new and old track slabs, as well as the pattern of load-bearing capacity deterioration. The conversion formula for the core sample concrete strength is as shown in Equation (1):

(1) $$f_{cu}^{c}=\alpha \frac{4F}{\pi d^{2}}$$

In this equation, $f_{cu}^{c}$ represents the core test piece converted to a 150 mm diameter cube compressive strength value (MPa); F represents the peak uniaxial compression test pressure of the drill core specimen; d represents the diameter of the drill core specimen; α represents the conversion coefficient of concrete strength corresponding to different height–diameter ratios of the drill core specimens, and 1.22 is taken in this experiment according to the specifications.

The peak loads of the 12 core samples from both new and old track slabs were substituted into the formula, allowing for the calculation of the cubic compressive strength for the new and old track slabs. The new track slab core samples exhibited an average peak load of 361.9 kN, corresponding to a cubic compressive strength of 56.2 MPa. In contrast, the old track slab core samples showed an average peak load of 280.7 kN, corresponding to a cubic compressive strength of 43.6 MPa.

The reduction in load-bearing capacity of the old slab is attributed to damage and deterioration, both on the surface and within the structure, resulting from fatigue loads. Compared to the new track slab, the compressive strength of the 10-year-old slab decreased by approximately 22.4%.

The initial defect locations within the new track slabs primarily stem from inadequate concrete compaction during vibration, as well as void damage caused by pressing or collisions during demolding and transportation. The extent of these voids is approximately 0.5%, indicating relatively good overall uniformity. Under the sustained effects of temperature and train loads, these defect locations will experience stress concentration first, leading to localized voids and cracking typical of damage accumulation processes. The overall damage of the track slab gradually extends along weak paths, with the overall void coefficient of the aged slabs reaching 3.6%. The increase in the internal void coefficient inevitably leads to issues such as decreased strength, degraded stiffness, and altered load transfer mechanisms, all of which affect the safe and stable operation of trains. This study aims to quantify the performance evolution of track slabs based on the number of voids, facilitating research into damage localization and evolution patterns under service conditions, and enabling the analysis of load-bearing capacity for slabs exceeding maintenance standards.

## 3. Modeling of Plastic Damage of Track Slab

### 3.1. Modeling Based on Concrete Plastic-Damage Coupling Theory

**Figure 4:**
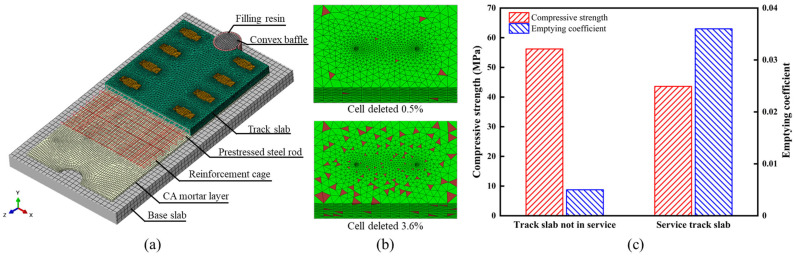
Performance comparison of old and new slabs and finite element simulation model. (**a**) Finite element analysis model of slab-type ballastless track. (**b**) Meshing comparison. (**c**) Corresponding relationship between compressive strength and emptying coefficient of new and old track slabs.

An analytical model comprising five track slabs was developed based on the concrete plastic-damage theory to examine the distribution and propagation of damage in ballastless track slabs containing internal void defects under coupled multi-field loads. As shown in Figure 4a, the model includes key components such as track slabs, mortar layers, base plates, protruding stoppers, filling resin, prestressed steel bars, and both transverse and longitudinal reinforcements. The track slab is a 4962-type prestressed flat slab, with dimensions of 4962 mm × 2400 mm × 190 mm. In the overall track slab model, the reinforcement and steel bars are modeled by two-node linear 3D truss elements (T3D2), while the mortar layer, base plate, protruding stoppers, and filling resin are simulated using eight-node linear hexahedral elements (C3D8R). The fasteners, assumed to have high stiffness, are modeled using four-node bilinear rigid quadrilateral elements (R3D4). While the track slab model for stress analysis uses four-node linear tetrahedral elements (C3D4), all other track slab models use eight-node linear hexahedral elements (C3D8R). According to the requirements of the concrete plastic-damage model, parameters such as the yield function, flow rule, and viscosity characteristics must be defined for the materials. The material parameters for the model are shown in Table 1.

To characterize the varying degrees of performance degradation in ballastless tracks during service, the void ratio was selected as an indicator to quantify the actual condition of track slabs at different service stages. As shown in Figure 4b, using the stochastic pore theory, the analytical track slab model was assigned different void ratios to simulate the damage evolution patterns at various stages of service.

### 3.2. Site Monitoring and Load Setting

Train loads and temperature loads are two major external environmental factors that significantly impact the stability and durability of ballastless track structures. When these factors act in different combinations, they can lead to various types of damage in the track structure. To obtain the characteristics of the train and temperature loads acting on the track slabs, corresponding tests and monitoring were conducted to capture the load distribution patterns.

**Figure 5:**
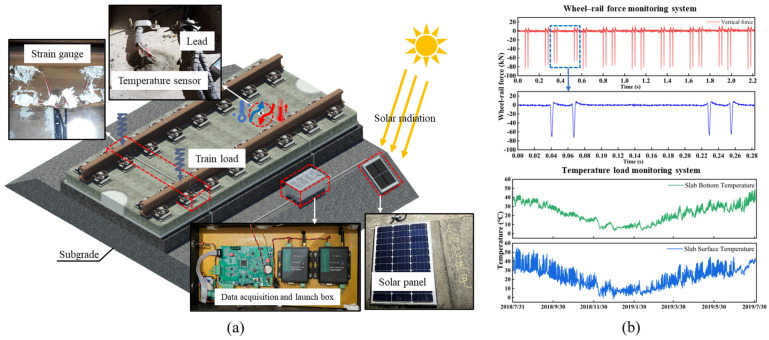
Field monitoring scheme. (**a**) Track online monitoring system. (**b**) Train load and temperature monitoring results.

For the measurement of wheel–rail forces, strain gauges were attached to the rails, and a dynamic resistance strain gauge system was used to capture the strain values in the rails under the influence of train loads. After calibration, the wheel–rail forces were calculated. The calibration process strictly adhered to the requirements of the “Ground Testing Methods for Lateral and Vertical Wheel-Rail Forces” (TB/T 2489-2016) [35]. Using a loading frame and loading device, lateral and vertical loads were applied to the rail in graded increments, and the corresponding strain responses were recorded. This process precisely established the mapping relationship between the applied loads and the strain signals. The results showed that the distribution characteristics of the wheel–rail forces generally followed a normal distribution, with the mean vertical wheel–rail force acting on the track slab being 84.0 kN. Temperature load, a key parameter affecting the stress state of ballastless track slabs, involves the combined effects of overall average temperature and nonlinear temperature gradients within the slab. For the measurement of track slab temperatures, temperature sensors were attached to the surface of the slab and embedded at corresponding locations inside the slab to capture the nonlinear distribution characteristics of the temperature field in real time. The data acquisition devices were powered by a solar energy system, and monitoring data were transmitted in real time to a back-end server via a wireless network. The temperature monitoring of the track slab was conducted over a period of one year. The recorded temperature variations between the surface and bottom of the track slab reached 60.9 °C and 39.8 °C, respectively, with the temperature gradient ranging from −50.4 °C/m to 100.0 °C/m, which exceeded the allowable limits specified in the design standards. The monitoring system is shown in Figure 5a and results are shown in Figure 5b.

To accurately simulate the service state of track slabs under the coupled effects of temperature and train-induced loads and to clarify their damage propagation characteristics, the long-term monitoring and inspection results from actual railway lines were appropriately extrapolated to generate the load spectrum for finite element simulation. Therefore, the load combination for scenario 1 was set to a combination of a wheel–rail force of 90.0 kN, an overall temperature rise of 35.0 °C, and a temperature gradient of 105.0 °C/m. The load combination for scenario 2 was set to a combination of a wheel–rail force of 90.0 kN, an overall temperature rise of 35.0 °C, and a temperature gradient of −55.0 °C/m. The load combination for scenario 3 was set to a combination of a wheel–rail force of 90.0 kN, an overall temperature drop of 35.0 °C, and a temperature gradient of 105.0 °C/m. The load combination for scenario 4 was set to a combination of a wheel–rail force of 90.0 kN, an overall temperature drop of 35.0 °C, and a temperature gradient of −55.0 °C/m.

### 3.3. Analysis of Surface Damage Evolution Law of Track Slab

**Figure 6:**
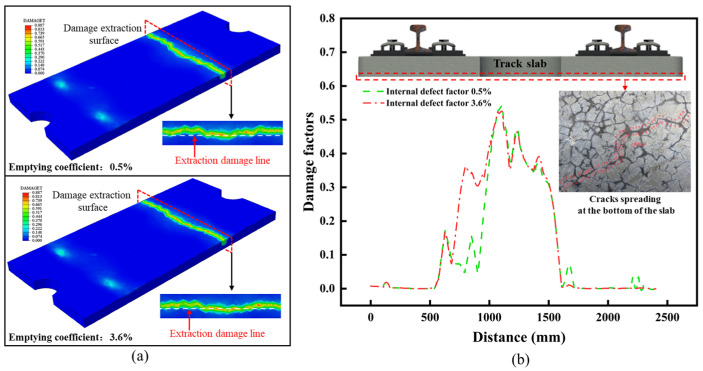
Simulation results of working condition 1. (**a**) Simulation results of old and new slabs under working condition 1. (**b**) Bottom damage extraction line and actual damage.

As shown in Figure 6a, under the load combination conditions of scenario 1, the maximum damage to the track slab occurred at the section directly under the train load, with the highest damage value reaching 0.887. As shown in Figure 6b, in both new and old structures, the maximum damage was located on the bottom surface of the track slab, representing a case of hidden damage. At the selected section, the damage value at the midspan of the bottom surface of the old track slab was generally higher than that of the new track slab. For prefabricated track slab structures, although curing conditions are effectively controlled, factors such as material properties, environmental conditions, and curing quality can still influence the slab. The upper surface of prefabricated concrete is most susceptible to cracking, and this results in initial cracking on the bottom surface of the track slab due to differences in temperature and humidity. Under the conditions of this scenario, fine cracks on the bottom surface of the track slab further propagate and extend, reducing the strength and durability of the track structure. Moreover, the damage propagation on the bottom surface weakens the bond with the underlying structural layers, accelerating the overall damage rate of the structure.

**Figure 7:**
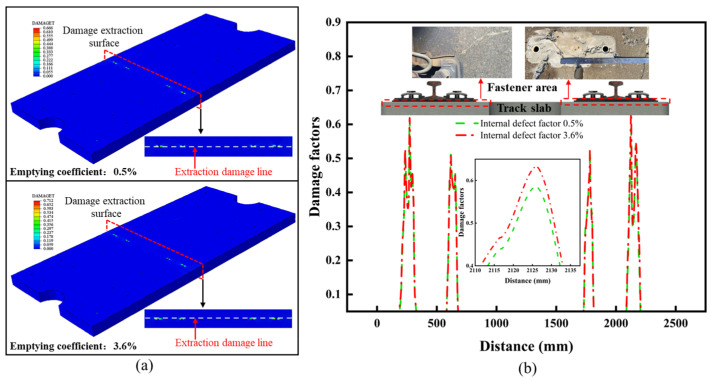
Simulation results of working condition 2. (**a**) Simulation results of old and new slabs under working condition 2. (**b**) Fastener area extraction line and actual damage.

As shown in Figure 7a, under the load combination conditions of scenario 2, the maximum damage to the track slab occurred in the region of the embedded insulating sleeve of the fasteners. The maximum damage value for the old track slab reached 0.712, representing a 6.9% increase compared to the new slab. As shown in Figure 7b, compared to other surface areas of the track slab, damage in the embedded insulating sleeve region is difficult to detect during routine inspections. As this region is critical for directly transmitting the wheel–rail forces to the track slab, any damage here can lead to stress concentration and a reduction in the supporting stiffness of the track slab.

**Figure 8:**
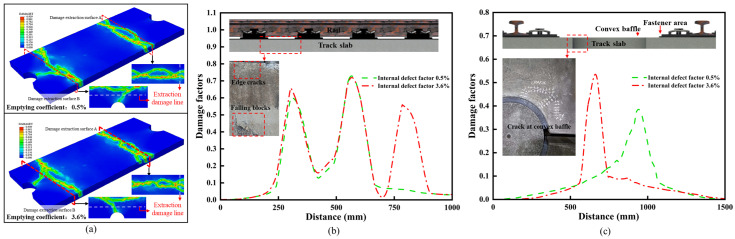
Simulation results of working condition 3: (**a**) Simulation results of old and new slabs under working condition 3; (**b**) Edge area extraction line and actual damage; (**c**) Boss area extraction line and actual damage.

As shown in Figure 8a, under the load combination conditions of scenario 3, the damaged area in the old track slab significantly increased compared to the new slab. In this scenario, there was a clear trend of damage propagating both longitudinally and transversely across the track slab, which is shown in Figure 8b. Longitudinally, as shown in Figure 8c, the damaged area in the old track slab gradually extended towards the region near the raised shoulder, forming a larger damage zone and leading to stress redistribution. Transversely, the damage spread from the edges of the fasteners towards the edge of the slab, eventually leading to through-thickness cracking. The slab ends and edge regions are prone to stress concentration effects and the cumulative impact of cyclic fatigue. The expansion of damage in these areas increases the risk of cracking in the track slab.

**Figure 9:**
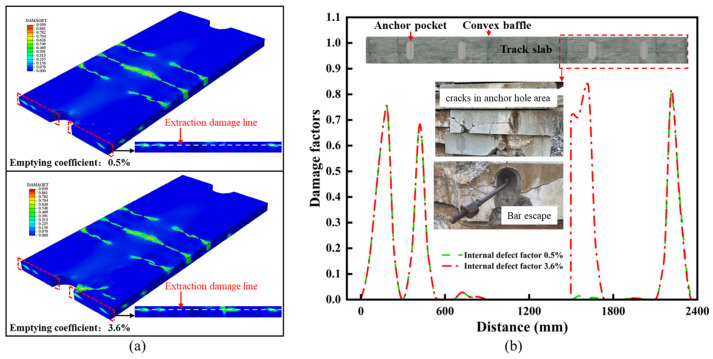
Simulation results of working condition 4. (**a**) Simulation results of old and new slabs under working condition 4. (**b**) Extraction line and actual damage in anchor hole area.

As shown in Figure 9a, under the load combination conditions of scenario 4, the maximum damage to the old track slab reached 0.939, with a significant increase in both the magnitude and area of damage in the anchorage region of the prestressed steel bars. As shown in Figure 9b, the anchor-age region of the track slab serves to protect the prestressed steel bars and is a critical area where new and old concrete bond, making it a weak point in the construction sequence. The deterioration and expansion of damage in the anchorage area increases the risk of local debonding, which can lead to the fracture or even displacement of the prestressed steel bars, ultimately affecting the durability of the track slab during service.

As the in-service performance of the track slab deteriorates, new damage areas tend to emerge along the edges of existing defects. These newly formed damage zones accumulate over time and continue to propagate, eventually leading to a comprehensive degradation of the track slab’s overall performance and a reduction in its load-bearing capacity. Essentially, this process involves alternating phases of damage initiation, accumulation, defect formation, and expansion. However, current research has not fully accounted for the changes in load transfer paths that occur once the track slab is damaged. Existing analyses often assume structural homogeneity, which does not reflect the reality of in-service deterioration. In fact, the degradation of the track slab alters the boundary conditions for damage propagation and the critical load thresholds, changing both the stress distribution and load transfer paths from the original design intent. This not only affects the actual in-service state of the structure but also triggers the emergence of new damage areas.

The analysis results indicate that the distribution of damage in the track slab varies under different load combination scenarios after overall performance degradation. In addition to shifts in damage extrema and locations, the damage propagation paths and affected areas change significantly. This is particularly evident in critical load-bearing regions such as fasteners, raised shoulders, and prestressed steel bar anchorages. These areas require special attention in routine maintenance and should be supplemented with reinforcement measures to mitigate further degradation.

## 4. Conclusions

To quantify the actual performance evolution and accurately evaluate the damage status of CRTS I track slabs in service, firstly, this study combined the impact-echo method and core drilling tests to obtain the variation laws of internal void and residual bearing capacity of the track slabs. Secondly, based on long-term monitoring and detection data of actual operating lines, the distribution characteristics and load values of temperature and wheel–rail force acting on the track slab were obtained. Finally, a CRTS I track slab analysis model with internal damage was established based on the concrete plasticity damage coupling model calculation theory. The damage distribution characteristics of the track slab after performance degradation under different load combinations were clarified. The main research conclusions are as follows:

1. The void coefficient inside the old track slab increased from 0.5% to 3.6%, and the expansion of internal void disease led to a decrease in the actual bearing strength of the track slab. The uniaxial compression test results of the core specimens showed that the average compressive strength of the track slab decreased from 56.2 MPa to 43.6 MPa after service, with a decrease of 22.4% in compressive strength.
2. Combining long-term monitoring and detection data of actual lines could provide data support for simulation analysis. The average wheel–rail force acting on the track slab was 84.0 kN. The temperature changes on the surface and bottom of the track slab reached 60.9 °C and 39.8 °C, respectively, and the temperature gradient range varied from −50.4 °C/m to 100.0 °C/m, exceeding the allowable value of the design specifications, which can easily lead to initial damage at critical stress locations.
3. Under different load combinations, there were significant differences in the damage distribution of track slabs after performance degradation. The damage distribution areas were mainly located near key stress positions such as fasteners, convex abutments, and anchor holes of prestressed steel bars. In addition to focusing on the extreme damage values and locations of track slabs under different load conditions, it is still necessary to accurately grasp the evolution of the damage propagation path of track slabs after overall performance degradation, in order to achieve the scientific maintenance and repair of track structures.

## Figures and Tables

**Table 1:** Material parameter selection of ballastless track model.

Components	Modulus of Elasticity/MPa	Poisson Ratio	Mass Density/kg m^−3^	Coefficient of Linear Expansion/°C^−1^
Slab	3.6 × 10^4^	0.2	2500	1.0 × 10^−5^
CA mortar layer	300	0.2	2000	1.8 × 10^−5^
Filling resin	-	0.1	1200	2.0 × 10^−5^
Convex baffle	3.3 × 10^4^	0.2	1200	1.0 × 10^−5^
Rebar	2.1 × 10^5^	0.3	7856	1.18 × 10^−5^

## Data Availability

The original contributions presented in this study are included in the article. Further inquiries can be directed to the corresponding author.
